# Usefulness of ^18^F-FDG PET/CT for evaluating response of ocular adnexal lymphoma to treatment

**DOI:** 10.1097/MD.0000000000010543

**Published:** 2018-04-27

**Authors:** Hayahiko Fujii, Hiroaki Tanaka, Yohei Nomoto, Naoki Harata, Sayako Oota, Jun Isogai, Katsuya Yoshida

**Affiliations:** a Department of Radiology; b Department of Hematology; c Department of Ophthalmology; d Department of Radiology; e PET Imaging Center, Asahi General Hospital, Asahi, Chiba, Japan.

**Keywords:** 18F-FDG, Lugano classification, ocular adnexal lymphoma, PET/CT, 5-point Deauville scale, treatment response

## Abstract

The purpose of this study was to investigate the efficacy of ^18^F-fluoro-deoxyglucose (FDG) positron emission tomography/computed tomography (PET/CT) for evaluating the ocular adnexal lymphoma treatment responses.

We retrospectively reviewed data for 9 histologically confirmed cases of malignant lymphoma. All patients had at least one ocular adnexal tumor site and underwent FDG PET/CT before and after treatment. Patients’ histological disease subtypes included diffuse large B-cell lymphoma (n = 3), mucosa-associated lymphoid tissue lymphoma (n = 2), follicular lymphoma (n = 1), NK/T-cell lymphoma (n = 1), lymphoplasmacytic lymphoma (n = 1), and Hodgkin lymphoma (n = 1). The highest FDG uptake by the ocular adnexal lesions was calculated as the maximum standardized uptake value (SUVmax). FDG uptake at ocular adnexal sites and sites of systemic disease after treatment were also assessed using the 5-point Deauville scale.

In 1 of the 9 patients, a conjunctival lesion could not be detected by either pre- or posttreatment PET/CT. For 8 of the 9 patients, the SUVmax value at the ocular adnexal site significantly decreased after treatment (7.1 ± 5.1 vs 1.6 ± 0.58; *P* = .0196). For 7 of the 9 patients, the first posttreatment FDG uptake at the ocular adnexal site was considered a complete metabolic response, and these patients showed an improved clinical ophthalmic presentation with no relapse at ocular adnexal sites during follow-up.

FDG PET/CT is useful for evaluation of the response of ocular adnexal lymphoma to treatment, although its usefulness may depend on the histological subtype and site of the lesion.

## 1 Introduction

Ocular adnexal lymphoma (OAL) accounts for approximately 1% of all non-Hodgkin lymphoma (NHL) and 15% of all extranodal NHL^[1]^, and is the most common malignant tumor in the ocular adnexa.^[2,3]^ OAL most often affects the lacrimal glands and intraorbital sites. About 7% to 24% of patients with OAL have lesions affecting the ocular adnexa bilaterally.^[1,3]^ The symptoms of OAL are frequently minor but vary depending on the ocular adnexal site involved and whether systemic disease is present. OAL consists of various histological subtypes of lymphoma, the majority of which are marginal zone B-cell lymphoma or mucosa-associated lymphoid tissue lymphoma.

^18^F-fluoro-deoxyglucose (FDG) positron emission tomography/computed tomography (PET/CT) is established for staging and assessment of response to treatment in patients with malignant lymphoma.^[4]^ The 5-point Deauville scale (5-PS) has been developed as a simple and useful method for representing different grades of FDG uptake.^[5]^ The 5-PS has been validated for the assessment of response in FDG-avid lymphoma and is recommended as the standard reporting method.^[6]^

English and Sullivan^[7]^ summarized the data from 8 studies that included a total of 130 patients with OAL and showed the sensitivity of FDG PET or PET/CT in detection of OAL. Recently, Thuro et al^[8]^ reported their rate of positive findings on FDG PET in the initial staging of 92 patients with OAL. However, to our knowledge, only 1 retrospective study and 3 case reports representing a total of 10 patients who underwent both pre- and posttreatment FDG PET or PET/CT scans have assessed the usefulness of this imaging modality in evaluation of the treatment response of OAL.^[9–12]^ Further, no study has assessed the treatment response of OAL using the 5-PS. The aim of this study was to investigate the usefulness of FDG PET/CT in evaluating the response of OAL to treatment using the 5-PS.

## 2 Materials and methods

### 2.1 Patients

The study was conducted with the approval of the ethics committee for clinical research at Asahi General Hospital. Written informed consent was waived for each patient for this study. We undertook a retrospective review of patient data for 5 men and 4 women (henceforth referred to as patients 1 through 9) of mean age 62 (range 25–86) years who had histologically confirmed malignant lymphoma with at least 1 ocular adnexal tumor site and underwent FDG PET/CT before and after treatment between January 2005 and December 2016. Patient clinical characteristics and treatment regimens are summarized in Tables 1 and 2. Patient ophthalmic presentations were as follows: swelling (n = 8), mass (n = 4), ocular motility disorder (n = 2), salmon patch (n = 1), proptosis (n = 2), and visual impairment (n = 1). Four patients presented with elevated serum total lactic dehydrogenase levels. Only patient 1 had B symptoms.

**Table 1:**
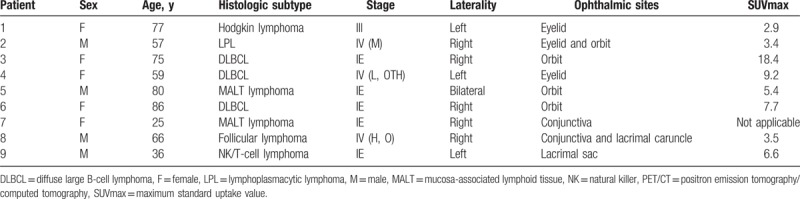
Patient characteristics and pretreatment PET/CT findings.

**Table 2:**
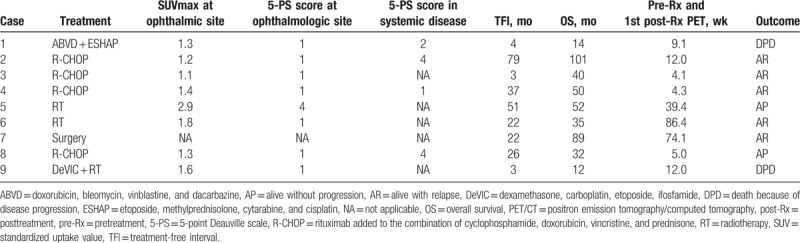
Posttreatment PET/CT findings, 5-PS scores, and outcome.

### 2.2 FDG PET/CT protocol

Images were acquired on a PET/CT scanner (Siemens Biograph Duo LSO, Siemens Medical Solutions USA Inc., Malvern, PA). CT studies for attenuation correction and anatomic coregistration were performed without contrast medium under free breathing. After the patient had fasted for at least 5 hours, FDG 3 MBq/kg was administered by intravenous injection. Whole body scanning was performed 100 minutes after FDG injection. PET data were acquired in 3-D mode on a 128 × 128 matrix (slice thickness, 5 mm). The acquisition time for PET imaging was 2 minutes per table position.

### 2.3 Image analysis

All acquired images were interpreted by 2 radiologists with 9 and 35 respective years of experience in nuclear medicine. Staging and assessment of response was based on the recommendations of the International Conference of Malignant Lymphomas Imaging Working Group.

Posttreatment FDG uptake was assessed using the 5-PS based on maximum intensity projection images (1, no uptake; 2, uptake lower than in the mediastinum; 3, uptake higher than in the mediastinum but lower than in the liver; 4, uptake moderately higher than in the liver; and 5, uptake markedly higher than in the liver).^[6]^

**Figure 1:**
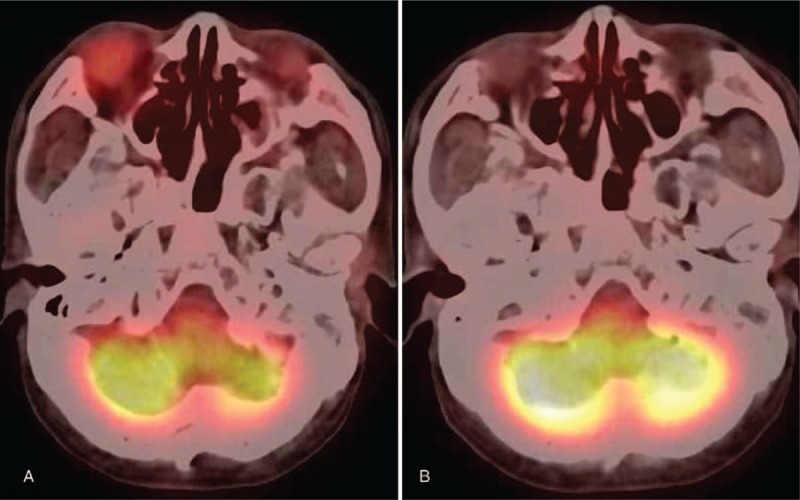
A 57-year-old man (patient 2) with lymphoplasmacytic lymphoma who had eyelid and orbital lesions. (A) Pretreatment PET/CT. (B) Posttreatment PET/CT. The pretreatment FDG uptake SUVmax was 3.4 in the right eyelid and orbital lesions. The posttreatment FDG uptake reduced to background level at the site (SUVmax 1.2). FDG = ^18^F-fluoro-deoxyglucose, PET/CT = positron emission tomography/computed tomography, SUVmax = maximum standardized uptake value.

We also drew a circular region of interest measuring 1 to 1.5 cm in diameter in the ocular adnexal lesion showing the highest FDG uptake to calculate the maximum standardized uptake value (SUVmax; Fig. 1).

### 2.4 Statistical analysis

A Student paired *t* test was used to compare pre- and posttreatment SUVmax values in the ocular adnexal lesion. These values were reported as mean ± standard deviations. Differences were assessed using a 2-sided analysis. *P*-values < .05 were considered statistically significant. All statistical analyses were performed using JMP statistical software v 10.0.2 (SAS Institute Inc., Cary, NC).

## 3 Results

Treatments, posttreatment PET/CT findings, 5-PS score, treatment-free intervals, overall survival data, and outcome data are summarized in Table 2. With the exception of patient 6, all patients underwent more than 1 posttreatment PET/CT examination. The SUVmax value at the ocular adnexal site significantly decreased after treatment (7.1 ± 5.1 vs 1.6 ± 0.58; *P* = .0196) for 8 of 9 patients (with the exception for patient 7), all of whom also showed an improved clinical ophthalmic presentation. Posttreatment FDG uptake at the ocular adnexal site was not increased when compared with the pretreatment uptake in any patient, and there were no reexacerbations of clinical ophthalmic presentation or relapses of systemic disease during follow-up.

For patient 5, the 1st posttreatment PET/CT had a score of 4 at the ocular adnexal site. This score decreased to 1 without additional treatment at the 2nd posttreatment PET/CT, which was performed 93.7 weeks after treatment.

For patient 7, the right conjunctival lesion could not be detected by either pre- or posttreatment PET/CT. Evaluation by an ophthalmologist indicated that this lesion had been growing slowly without reexacerbation of the patient's clinical ophthalmic presentation and the patient underwent radiation therapy.

Patients 1, 2, 3, 4, 6, and 12 underwent 2nd-line treatment for relapse of systemic disease. Patients 1, 2, and 4 showed increased FDG uptake in residual systemic lesions on 2nd or further post-PET/CT. Patients 3, 6, and 9 (initial stage IE) relapsed with new systemic lesions, which were also detected by posttreatment PET/CT.

## 4 Discussion

Our study suggests that PET/CT is useful for evaluating the response of OAL to treatment. On average, 1st posttreatment FDG uptake at ocular adnexal sites was lower than the pretreatment uptake in 8 (88%) of 9 patients and corresponded to improved clinical ophthalmic presentation. Our findings are consistent with those of 4 previous reports.^[9–12]^ However, differences in the histological subtypes of the cases analyzed between this and the previous reports validate the broader application of PET/CT. In the 4 previous reports, histological subtypes comprised mucosa-associated lymphoid tissue (n = 6), large-cell lymphoma (n = 2), lymphoid hyperplasia (n = 1), and follicular lymphoma (n = 1). In contrast, the histological subtypes observed in our study comprised Hodgkin lymphoma (HL) (n = 1), lymphoplasmacytic lymphoma (n = 1), NK/T-cell lymphoma (n = 1), and diffuse large B-cell lymphoma (DLBCL) (n = 3), which were not represented in the previous reports. Therefore, our results may support the usefulness of FDG PET/CT when evaluating the response of these OAL subtypes to treatment. Further, the mean follow-up duration in our study (47.2 months) was longer than that in the previous retrospective report (7.6 months).^[9]^ Our study also included multiple posttreatment PET/CT in 8 of 9 patients, whereas only 1 patient underwent multiple posttreatment PET/CT in the previous reports. These observations may be reflected by increased accuracy of the results obtained in our study.

Using the 5-PS, posttreatment FDG uptake at the ocular adnexal site was rated as a complete metabolic response (score 1) in 7 of 9 patients. These assessments are appropriate from a clinical point of view and for long-term follow-up. Regarding assessment of the response of systemic lesions, in 2 of 4 patients with systemic disease at the initial staging posttreatment FDG uptake by systemic lesions were considered a partial metabolic response (score 4). However, in the remaining 2 of the 4 patients, the posttreatment FDG uptake by systemic lesions was judged to be a complete metabolic response (scores 1–2). These 2 patients had reexacerbation of their lesions after the 1st posttreatment PET/CT. In view of the small size of our study and the variable histologic subtypes of OAL, further studies are needed to confirm the effectiveness of the 5-PS. In particular, statistical analyses, such as positive predictive and negative predictive values, should be used to evaluate the response of OAL to therapy.

For patient 5, FDG uptake in the 2nd posttreatment PET/CT at the ocular adnexal sites was lower compared to their 1st posttreatment PET/CT (with a decrease in score from 4 to 1). Notably, this patient had undergone radiation therapy prior to the 1st posttreatment PET/CT. Transient radiation-induced inflammation may have caused this increased uptake, although the interval between completion of radiation therapy and the 1st posttreatment PET/CT was longer than the recommended interval of 8 to 12 weeks.^[13]^

Patient 7 had a conjunctival lesion that could not be detected by either pre- or posttreatment FDG PET/CT, nor MRI, because of the small size of the lesion and the low-grade of the tumor. Zanni et al^[14]^ previously reported that the sensitivity of FDG PET for conjunctival lymphoma is limited to 35%.

There are some limitations to our study, in particular, its retrospective design and small patient sample size. Additionally, the patients included in the study were evaluated over a long period (11 years), although there was no change in the PET/CT scan acquisition protocol during this time. However, it should be noted that there are differences in the resolution and sensitivity between our PET/CT scanner and present state-of-the art PET/CT scanning devices that may have influenced our detection rates and the SUVmax.

In conclusion, FDG-PET/CT is useful for evaluating the response of OAL to treatment. However, this method may be affected by histologic subtype and site of the lesion.

## Acknowledgments

The authors thank Dr Yasunori Sato of the Clinical Research Center, Chiba University Hospital, Chiba, Japan, for his advice concerning statistical analysis.

## Author contributions

**Conceptualization:** Hayahiko Fujii.

**Data curation:** Hayahiko Fujii.

**Formal analysis:** Hayahiko Fujii.

**Investigation:** Hayahiko Fujii.

**Project administration:** Hayahiko Fujii.

**Resources:** Hayahiko Fujii.

**Supervision:** Hayahiko Fujii, Hiroaki Tanaka, Yohei Nomoto, Naoki Harata, Sayako Oota, Jun Isogai, Katsuya Yoshida.

**Validation:** Hayahiko Fujii, Hiroaki Tanaka, Yohei Nomoto, Naoki Harata, Sayako Oota, Jun Isogai, Katsuya Yoshida.

**Writing – original draft:** Hayahiko Fujii.

**Writing – review & editing:** Hayahiko Fujii.
